# Man With Chest Pain and Unilateral Weakness

**DOI:** 10.1016/j.acepjo.2025.100186

**Published:** 2025-05-25

**Authors:** Holly Ryan, Molly Hartrich, Wesley Eilbert

**Affiliations:** Department of Emergency Medicine, University of Illinois, College of Medicine, Chicago, Illinois, USA

**Keywords:** left ventricular thrombus, ventricular thrombus, ultrasound ventricular thrombus, myocardial infarction ventricular thrombus, ventricular thrombus embolic stroke, myocardial infarction stroke

## 1 Patient Presentation

**Figure 1:**
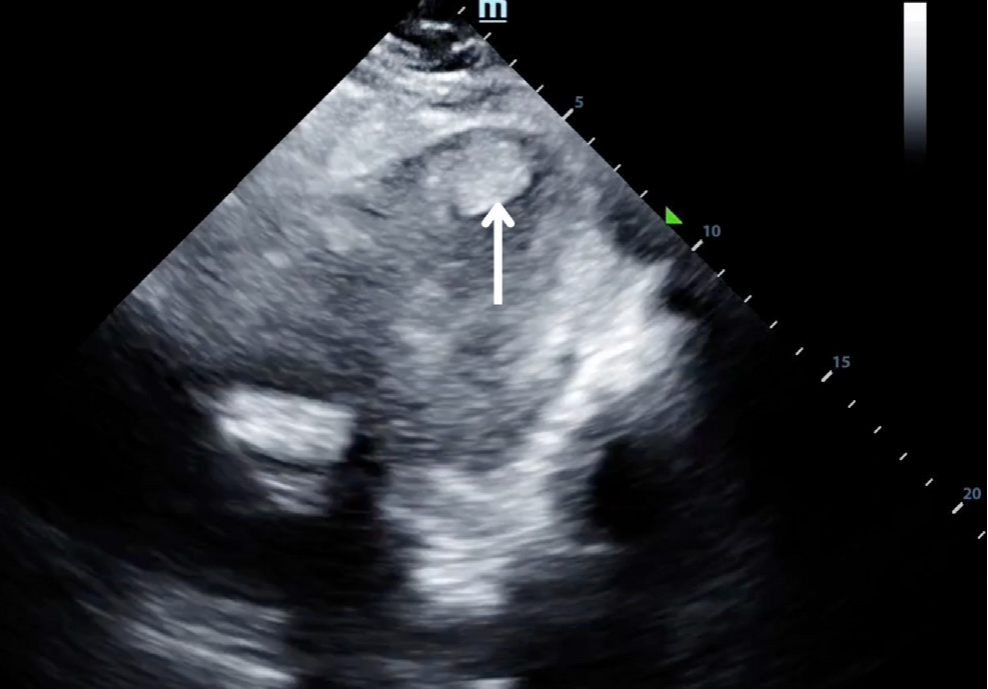
Sonographic apical cardiac view demonstrating a hyperechoic thrombus (arrow) in the apex of the left ventricle.

**Figure 2:**
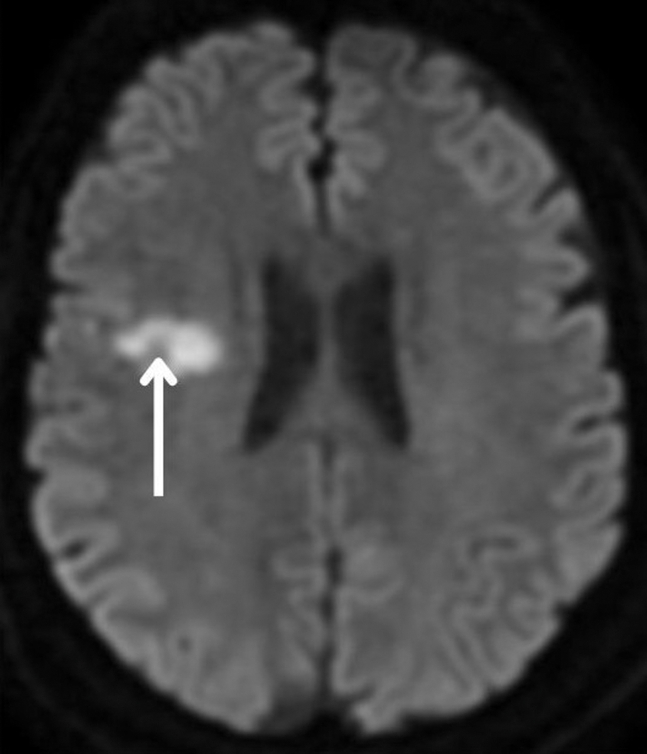
Magnetic resonance imaging revealing high signal intensity in the right frontal lobe (arrow), consistent with acute ischemia.

A 48-year-old man with a history of diabetes mellitus and hypertension presented to the emergency department with 1 day of anterior chest pain. Shortly after the onset of chest pain, he noticed weakness in his left arm and leg. Physical examination was notable for weakness of the left arm and leg compared with the right. His electrocardiogram demonstrated ST elevations in leads V3 to V6, and his serum troponin I level was markedly elevated. Bedside cardiac ultrasonography (Fig 1 and Video) and magnetic resonance imaging of the brain (Fig 2) were performed.

**Video:**
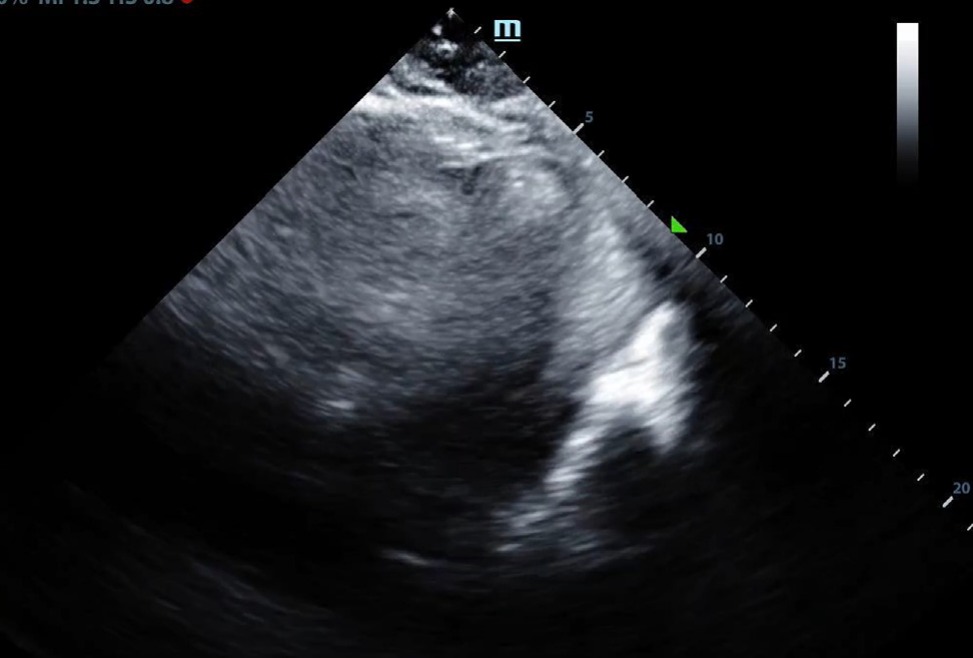
Left ventricular thrombus.

## 2 Diagnosis: Acute Myocardial Infarction Complicated by Left Ventricular Thrombus with Embolic Ischemic Stroke

Although the incidence of left ventricular thrombus (LVT) after myocardial infarction has decreased because of modern reperfusion therapies, 6.3% of ST elevation myocardial infarctions will develop an LVT.^1^ Myocardial infarctions at increased risk for LVT formation include those with a large area of infarction or involving the anterior wall or left ventricular apex, those with left ventricular dyskinesis or reduced ejection fraction, and those with severe diastolic dysfunction.^2^^,^^3^

Although contrast-enhanced cardiovascular magnetic resonance imaging is the most sensitive imaging modality for LVT, bedside cardiac ultrasound is useful for rapid diagnosis in the emergency department.^4^ Untreated, the annual rate of stroke or other systemic embolization events with LVT is 10% to 15%, with the risk of LVT-related stroke after a myocardial infarction being highest in the first 4 days.^5^^,^^6^ Anticoagulation with either vitamin K antagonists or direct oral anticoagulants significantly reduces the risk of systemic embolization with LVT.^5^

## Funding and Support

By *JACEP Open* policy, all authors are required to disclose any and all commercial, financial, and other relationships in any way related to the subject of this article as per ICMJE conflict of interest guidelines (see www.icmje.org). The authors have stated that no such relationships exist.

## References

[1] Camaj A., Fuster V., Gustino G. (2022). Left ventricular thrombus following acute myocardial infarction: JACC state-of-the-art review. J Am Coll Cardiol.

[2] Bulluck H., Chan M.H.H., Paradies V. (2018). Incidence and predictors of left ventricular thrombus by cardiovascular magnetic resonance in acute ST-segment elevation myocardial infarction treated by primary percutaneous coronary intervention: a meta-analysis. J Cardiovasc Magn Reson.

[3] Cruz Rodriguez J.B., Okajima K., Greenberg B.H. (2021). Management of left ventricular thrombus: a narrative review. Ann Transl Med.

[4] Patel M., Wei X., Weigel K. (2021). Diagnosis and treatment of intracardiac thrombus. J Cardiovasc Pharmacol.

[5] Levine G.N., McEvoy J.W., Fang J.C. (2022). Management of patients at risk for and with ventricular thrombosis: a scientific statement from the American Heart Association. Circulation.

[6] Fonseca A.C. (2023). Stroke and recent myocardial infarction, reduced ejection fraction, left ventricular thrombus, and wall motion abnormalities. Curr Cardiol Rep.

